# Supplementary material for: Protocol for the development of a core outcome set for studies of pregnant women with pre-existing multimorbidity
Source: BMJ Open. 2021 Oct 29;11(10):e044919. doi: 10.1136/bmjopen-2020-044919 (PMC8559099; doi:10.1136/bmjopen-2020-044919)
Supplement: Supplementary data [file bmjopen-2020-044919supp001.pdf]

Appendix 1: The Core Outcome Set-STAndardised Protocol Items (COS-STAP) Statement

Sections	No	Items	Location in manuscript
TITLE/ABSTRACT			
Title	1a	Identify in the title that the paper describes the protocol for the planned development of a COS	page 1 Title page
Abstract	1b	Provide a structured abstract	page 2 Abstract
INTRODUCTION			
Background and objectives	2a	Describe the background and explain the rationale for developing the COS, and identify the reasons why a COS is needed and the potential barriers to its implementation	page 4 Background
	2b	Describe the specific objectives with reference to developing a COS	page 4 Background
Scope	3a	Describe the health condition(s) and population(s) that will be covered by the COS	Page 5: Scope of the COS
	3b	Describe the intervention(s) that will be covered by the COS	Page 5: Scope of the COS
	3c	Describe the context of use for which the COS is to be applied	Page 5: Scope of the COS
METHODS			
Stakeholders	4	Describe the stakeholder groups to be involved in the COS development process, the nature of and rationale for their involvement and also how the	Table 1 and Table 2

		individuals will be identified; this should cover involvement both as members of the research team and as participants in the study	
Information sources	5a	Describe the information sources that will be used to identify the list of outcomes. Outline the methods or reference other protocols/papers	Page 5: Stage 1 Systematic literature search<br>Page 6: Stage 2 Focus groups
	5b	Describe how outcomes may be dropped/combined, with reasons	Page 8: Initial list of outcomes
Consensus process	6	Describe the plans for how the consensus process will be undertaken	Page 11: Consensus meeting
Consensus definition	7a	Describe the consensus definition	Page 10: Score criteria for consensus
	7b	Describe the procedure for determining how outcomes will be added/combined/dropped from consideration during the consensus process	Page 10: Score criteria for consensus
ANALYSIS			
Outcome scoring/feedback	8	Describe how outcomes will be scored and summarised, describe how participants will receive feedback during the consensus process	Page 10: 1st-3rd Delphi survey
Missing data	9	Describe how missing data will be handled during the consensus process	Page 10: 3rd Delphi survey
ETHICS and DISSEMINATION			
Ethics approval/informed consent	10	Describe any plans for obtaining research ethics committee/institutional review board approval in relation to the consensus process and describe	Page 6: Focus group<br>Page 8: 1st Delphi<br>Page 12: Ethics

		how informed consent will be obtained (if relevant)	
Dissemination	11	Describe any plans to communicate the results to study participants and COS users, inclusive of methods and timing of dissemination	Page 12: Dissemination
ADMINISTRATIVE INFORMATION			
Funders	12	Describe sources of funding, role of funders	Page 12: Funding statement
Conflicts of interest	13	Describe any potential conflicts of interest within the study team and how they will be managed	Page 12: Conflict of interest
