# Supplementary material for: Protocol for the development of a core outcome set for studies of pregnant women with pre-existing multimorbidity
Source: BMJ Open. 2021 Oct 29;11(10):e044919. doi: 10.1136/bmjopen-2020-044919 (PMC8559099; doi:10.1136/bmjopen-2020-044919)
Supplement: Author's manuscript [file bmjopen-2020-044919.draft_revisions.pdf]

BMJ Open is committed to open peer review. As part of this commitment we make the peer review history of every article we publish publicly available.

When an article is published we post the peer reviewers' comments and the authors' responses online. We also post the versions of the paper that were used during peer review. These are the versions that the peer review comments apply to.

The versions of the paper that follow are the versions that were submitted during the peer review process. They are not the versions of record or the final published versions. They should not be cited or distributed as the published version of this manuscript.

BMJ Open is an open access journal and the full, final, typeset and author-corrected version of record of the manuscript is available on our site with no access controls, subscription charges or pay-per-view fees (http://bmjopen.bmj.com).

If you have any questions on BMJ Open's open peer review process please email info.bmjopen@bmj.com

BMJ Open

Core outcome set for studies of pregnancy affected by multimorbidity: a protocol

Journal:	*BMJ Open*
Manuscript ID	bmjopen-2020-044919
Article Type:	Protocol
Date Submitted by the Author:	19-Sep-2020
Complete List of Authors:	Lee, Siang Ing; University of Birmingham Institute of Applied Health Research,<br>Eastwood, Kelly-Ann; Queen's University Belfast, Centre for Public Health<br>Moss, Ngawai; Patient and Public Representative<br>Azcoaga-Lorenzo, Amaya; University of St Andrews, Division of Population and Behavioural Sciences, School of Medicine<br>Subramanian, Anuradha; University of Birmingham Institute of Applied Health Research<br>Anand, Astha; University of Birmingham Institute of Applied Health Research<br>Taylor, Beck; University of Birmingham Institute of Applied Health Research<br>Nelson-Piercy, Catherine; Guy's and Saint Thomas' NHS Foundation Trust<br>Yau, Christopher; The University of Manchester, Division of Informatics, Imaging and Data Sciences, Faculty of Biology Medicine and Health<br>McCowan, Colin ; University of St Andrews, School of Medicine<br>OReilly, Dermot; Queen's University Belfast, Epidemiology and Public Health<br>Hope, Holly; The University of Manchester Faculty of Biology Medicine and Health, Centre for Women's Mental Health, Division of Psychology and Mental Health, School of Health Sciences<br>Kennedy, Jonathan; Swansea University College of Medicine<br>Abel, Kathryn ; The University of Manchester Faculty of Biology Medicine and Health, Centre for Women's Mental Health, Division of Psychology and Mental Health; Greater Manchester Mental Health NHS Foundation Trust<br>Locock, Louise; University of Aberdeen, Health Service Research<br>Brocklehurst, Peter; University of Birmingham Institute of Applied Health Research<br>Plachcinski, Rachel; Patient and Public Representative<br>Brophy, Sinead; Swansea University College of Medicine<br>Agrawal, Utkarsh; University of St Andrews, School of Medicine<br>Thangaratinam, Shakila; University of Birmingham, Institute of Metabolism and Systems Research<br>Nirantharakumar, Krishnarajah; University of Birmingham Institute of Applied Health Research<br>Black, Mairead; University of Aberdeen, School of Medicine, Medical Science and Nutrition
Keywords:	Maternal medicine < OBSTETRICS, PUBLIC HEALTH, PREVENTIVE

	MEDICINE

SCHOLARONE™
Manuscripts

I, the Submitting Author has the right to grant and does grant on behalf of all authors of the Work (as defined in the below author licence), an exclusive licence and/or a non-exclusive licence for contributions from authors who are: i) UK Crown employees; ii) where BMJ has agreed a CC-BY licence shall apply, and/or iii) in accordance with the terms applicable for US Federal Government officers or employees acting as part of their official duties; on a worldwide, perpetual, irrevocable, royalty-free basis to BMJ Publishing Group Ltd ("BMJ") its licensees and where the relevant Journal is co-owned by BMJ to the co-owners of the Journal, to publish the Work in this journal and any other BMJ products and to exploit all rights, as set out in our licence.

The Submitting Author accepts and understands that any supply made under these terms is made by BMJ to the Submitting Author unless you are acting as an employee on behalf of your employer or a postgraduate student of an affiliated institution which is paying any applicable article publishing charge ("APC") for Open Access articles. Where the Submitting Author wishes to make the Work available on an Open Access basis (and intends to pay the relevant APC), the terms of reuse of such Open Access shall be governed by a Creative Commons licence – details of these licences and which Creative Commons licence will apply to this Work are set out in our licence referred to above.

Other than as permitted in any relevant BMJ Author's Self Archiving Policies, I confirm this Work has not been accepted for publication elsewhere, is not being considered for publication elsewhere and does not duplicate material already published. I confirm all authors consent to publication of this Work and authorise the granting of this licence.

For peer review only

Title: Core outcome set for studies of pregnancy affected by multimorbidity: a protocol

Authors: Siang Ing Lee,[1] Kelly-Ann Eastwood,*[2] Ngawai Moss*, Amaya Azcoaga-Lorenzo,[3] Anuradhaa Subramanian,[1] Astha Anand,[1] Beck Taylor,[1] Catherine Nelson-Piercy,[4] Christopher Yau,[5] Colin McCowan,[3] Dermot O'Reilly,[2] Holly Hope,[6] Jonathan I Kennedy,[7] Kathryn Abel,[6, 8] Louise Locock,[9] Peter Brocklehurst,[1] Rachel Plachcinski, Sinead Brophy,[7] Utkarsh Argawal,[3] Shakila Thangaratinam,*[10] Krishnarajah Nirantharakumar,*[1] Mairead Black*[9]

* indicates equal contribution

Affiliation:

1. Institute of Applied Health Research, University of Birmingham, Birmingham, UK
2. Centre for Public Health, Queen's University of Belfast
3. School of Medicine, University of St Andrews, UK
4. Guy's and St. Thomas' NHS Foundation Trust
5. Division of Informatics, Imaging and Data Sciences, Faculty of Biology Medicine and Health, The University of Manchester
6. Centre for Women's Mental Health, Division of Psychology and Mental Health, School of Health Sciences, Faculty of Biology Medicine & Health, The University of Manchester, UK
7. Data Science, Medical School, Swansea University, UK
8. Greater Manchester Mental Health NHS Foundation Trust, Manchester
9. School of Medicine, Medical Science and Nutrition, University of Aberdeen, UK
10. Institute of Metabolism and Systems Research, University of Birmingham, Birmingham, UK

Authors ORCID: Siang Ing Lee 0000-0002-2332-5452, Kelly-Ann Eastwood 0000-0003-3689-0490, Ngawai Moss 0000-0001-9369-5072, Amaya Azcoaga-Lorenzo 0000-0003-3307-878X, Anuradhaa Subramanian 0000-0001-8875-7363, Astha Anand 0000-0003-0494-321X, Beck Taylor 0000-0002-3559-7922, Catherine Nelson-Piercy 0000-0001-9311-1196, Christopher Yau 0000-0001-7615-8523, Colin McCowan 0000-0002-9466-833X, Dermot O'Reilly 0000-0002-9181-0652, Holly Hope 0000-0002-4834-6719, Jonathan I Kennedy, Kathryn Abel 0000-0003-3538-8896, Louise Locock 0000-0002-8109-1930, Peter Brocklehurst 0000-0002-9950-6751, Rachel Plachcinski 0000-0001-9908-0773, Sinead Brophy 0000-0001-7417-2858, Utkarsh Argawal 0000-0001-5181-6120, Shakila Thangaratinam 0000-0002-4254-460X, Krishnarajah Nirantharakumar 0000-0002-6816-1279, Mairead Black 0000-0002-6841-8601

Corresponding author: Krishnarajah Nirantharakumar, K.Nirantharan@bham.ac.uk,

IOEM Building, University of Birmingham, Edgbaston, Birmingham, B15 2TT, UK

Word count: 2447

Figures: 2

Appendix: 1

Keywords: multimorbidity, pregnancy, core outcome set

For peer review only - http://bmjopen.bmj.com/site/about/guidelines.xhtml

ABSTRACT

Introduction

Increasingly more pregnant women are living with pre-existing multimorbidity and this may affect maternal and offspring outcomes. Further research is required to understand this association and to develop interventions. However, there is currently no existing core outcome set (COS) for this area of research.

Methods and analysis

This study aims to develop a COS for maternal (antenatal, intra-partum, post-partum, long-term) and offspring (newborn, neonatal, infant, prepubertal, pubertal, early adulthood) outcomes in pregnant women affected by pre-existing multimorbidity.

An initial list of outcomes will be identified through a hierarchical systematic literature search of over 90 morbidities chosen due to their potential effect on pregnancy. These morbidities were identified by the scientific advisory group (including patient representatives) and will be grouped into disease categories. The literature search will be in the following order for each morbidity of interest: published COS, systematic reviews in parallel with patient reported outcome measures studies, and primary studies (observational/interventional).

The COS will be selected through a three round online Delphi survey followed by a consensus meeting. The stakeholders will include women (and/or their partners) with experience of pregnancy in the last three years who have multimorbidity, health/social care professionals involved in their care and researchers in this field. Stakeholders will predominantly be from the United Kingdom.

Ethics and dissemination

We will seek required ethical approval prior to the consensus setting process. The final COS will be disseminated through peer-reviewed publication and conferences and to all stakeholders.

Strengths and limitations of this study

- COS development in accordance to the COS-STAD
- Extensive patient, public and stakeholder involvement at each stage
- Pragmatic design to make covering multiple component diseases (contributing to multimorbidity) feasible
- Final COS separated into main set for all pregnancies and may consider disease specific subsets to allow flexible use in research
- Literature search limited to English language

For peer review only - http://bmjopen.bmj.com/site/about/guidelines.xhtml

BACKGROUND

Multimorbidity refers to a state of having two or more long-term physical or mental health conditions.[1] It is becoming more common in women of reproductive age.[2 3] As these women enter pregnancy, the pre-existing multimorbidity may have short and long term effects on them, their pregnancy and their offspring; and complex care needs can arise.[4 5] For example, rates of preterm birth are almost three times higher in women with multimorbidity (15 per 100 in hospital deliveries).[4] Over 80% of maternal deaths occur in women with multimorbidity or complex social factors.[6] There is a recognised need for national guidance on the management of women with multimorbidity before, during and after pregnancy.[6]

To understand the association between multimorbidity and pregnancy outcomes, researchers first need to develop a core outcome set (COS). COS is a set of outcomes that is expected to be reported in research as a minimum standard.[7] COS may reduce heterogeneity between clinical studies, allow for evidence synthesis and reduce selective reporting bias.[7] It is developed with stakeholders including patients, health care professionals and researchers through a consensus setting process. The Core Outcome Measures in Effectiveness Trial (COMET) initiative collates resources for COS development and maintains a COS database.[8] Although COS for studies of pregnancy and single disorders exist, there is currently none for pregnancy with multimorbidity.[8]

This study aims to develop a maternal and offspring COS for studies of pregnancy affected by multimorbidity. A major challenge is the vast number of single diseases that can contribute to multimorbidity. Therefore, a pragmatic study design is required to make the process feasible and manageable.

The proposed COS will be applicable for all clinical research. Further interventional studies are urgently needed to tackle multimorbidity in pregnancy and reduce the associated adverse outcomes. It is therefore important to have a predefined COS to inform future research studies to enable valid comparisons between study findings.

METHODS

This study is designed in accordance with the COS standards for development (COS-STAD) recommendations; study findings will be reported following the COS standards for reporting (COS-STAR).[7 9 10]

The study will consist of three stages: 1) hierarchical systematic search of the literature to identify maternal and offspring outcomes; 2) Delphi surveys amongst stakeholders to prioritise the core outcomes; and 3) a consensus meeting to agree on the final COS (Figure 1).

For peer review only - http://bmjopen.bmj.com/site/about/guidelines.xhtml

Scope of the COS

The population is pregnant women; the exposure is pre-existing multimorbidity. The COS would be applicable principally to observational studies but can inform interventional studies as well for pregnancy in all settings.

Maternal outcomes will include antenatal, intrapartum, post-partum and long-term outcomes. Offspring outcomes will include the newborn (first seven days), neonatal (first one month), infant (first one year), pre-pubertal (two to 11 years old), pubertal period (12-18 years old) and adulthood.[11] Pregnancy outcomes in the rest of this protocol will refer to both maternal and offspring outcomes.

Patient and public involvement (PPI)

PPI for this study will be three-tiered: (1) patient representatives in the scientific advisory group (SAG), (2) PPI advisory group and (3) patient and public stakeholders as research participants.

The SAG consists of clinicians, researchers and patient co-applicants collaborating on a larger project investigating pregnancy with multimorbidity (MuM-PreDiCT).[12] PPI co-applicant NM has advised on the study design, co-authored this protocol and created Figure 2 that illustrates the PPI in the COS development.[13]

Stakeholders for COS (participants of Delphi surveys and consensus meeting)

Three main groups of stakeholders will be invited to participate in the consensus setting process using various recruitment channels and snowballing. The stakeholders will be predominantly limited to the United Kingdom (UK). Participants have to be aged 18 years or older.

i. Patient and public representatives

Women with an experience of pregnancy within the last three years and have two or more pre-existing morbidities and/or their partners or carers will be eligible. Participants will be recruited from a wide range of antenatal clinics, maternity service user associations, parent support networks, relevant charities and social media, to ensure the representativeness and diversity of the sample. Care will be taken in explaining the concept of COS to lay participants, using supporting materials from the COMET website.[8]

ii. Health and social care professionals

This will include obstetricians, physicians, paediatricians, neonatologists, primary care clinicians, public health professionals and any health or social care professionals involved in providing multidisciplinary team care for pregnant women (for example, clinicians of established joint antenatal clinics, perinatal mental health team, drug and alcohol services, social services, midwives, health visitors, dieticians, et cetera). Healthcare professionals will

For peer review only - http://bmjopen.bmj.com/site/about/guidelines.xhtml

be recruited through NHS trusts that the research collaborators are affiliated with, contacts of research collaborators and professional societies.

iii. Researchers

This will include researchers and journal editors with an interest in pregnancy with multimorbidity.

Stage 1: Hierarchical systematic literature search

An initial list of outcomes will be identified through a hierarchical systematic literature search. Multimorbidity consists of individual health conditions and can encompass different combinations. The SAG prioritised over 90 morbidities to be studied, though future multimorbidity studies depending on specific question may exclude some and instead incorporate others. Relevant morbidities may be grouped into categories, for example, mental health conditions or cardiovascular disease, to make the literature search feasible.

For each morbidity / disease category, a hierarchical literature search by study design will be conducted in the following order of priority, as required:

1. Published COS[14]
2. In parallel:
   a. systematic reviews of observational/interventional studies[15 16]
   b. patient reported outcome measures (PROM) studies[17]
3. Primary observational /interventional studies[18]

For each health condition, if literature already exists at the top of the hierarchy, this will be used to obtain the initial list of outcomes and no further search is required.

Search strategy

The following databases will be searched: COMET database (COS), Cochrane library (systematic reviews) and Medline (systematic reviews, PROM and primary studies). The COMET database is updated regularly with an annual systematic review search of Medline, SCOPUS and Cochrane Methodology Register.[19 20]

Relevant key search terms will include pregnancy (population), relevant morbidity (exposure) and relevant study design. Appendix 1 presents example search strategies.

No time limits will be applied for the search of existing COS.[7] Literature search for other study designs will be conducted in stages, tracking retrospectively year by year, until data saturation is reached (no further unique outcome is identified).[7 21] For primary studies, the search will be discontinued if no eligible studies have been identified for three consecutive years, experts in the field will be consulted to identify any missing papers and the reference list of included studies will be screened. The search strategy may be modified as we learn from the initial exploration of the literature.

For peer review only - http://bmjopen.bmj.com/site/about/guidelines.xhtml

Study selection and data extraction

The inclusion criteria are: study design according to the hierarchy, studies reporting pregnancy outcomes, exposure morbidities/disease categories of interest and published in English. The exclusion criteria are: ongoing studies with no published outcomes, narrative reviews, case reports, case series, diagnostic accuracy studies, laboratory studies and animal studies. Full text screening will be conducted by two independent reviewers.

Two reviewers will extract the following data from included studies: author, year of publication, study design, exposure morbidities/disease categories, PROM domains, types of pregnancy outcomes and definition of outcome.

Any discrepancy between the two independent reviewers for study selection and data extraction will be resolved with a third reviewer.

Initial list of outcomes

The initial list of outcomes will be reviewed and refined by the SAG and PPI advisory group. Similar outcomes may be combined.[7 14] Pregnancy outcomes will be categorised into: (i) a main set that is common across all pregnancies and (ii) outcomes specific to individual morbidities/disease categories.

Stage 2: Delphi survey

The Delphi technique collates stakeholder opinions using sequential surveys. The response is summarised and fed back to stakeholders in subsequent rounds. Stakeholders consider the collective views before re-rating the outcomes. This provides a mechanism to reconcile different opinions to reach a consensus.[7] This study will employ a three round Delphi survey which is generally sufficient to reach consensus (Figure 1).[22]

The surveys will be hosted on a secure platform online. Participant characteristics including socio-demographics, specialty and job roles (health care professionals and researchers), disease categories (patients) will be requested. Participant's name and email contact will be included to avoid duplicate entry, for sending up to two personalised reminders (one week apart) and following up on incomplete response. This information will be kept separate from the survey responses.

The wording of the survey will be developed with the SAG and PPI advisory group to ensure plain language is used to describe the outcomes. Outcomes will be presented in alphabetical order to avoid any response effects related to the order of survey items.[7 21]

Each outcome will be rated on a 9 point Likert scale: 1-3 (not important), 4-6 (important but not critical) and 7-9 (critically important). An '*unable to score'* option will be provided to allow for participants who may not have the expertise to score certain outcomes.[7] The 9 point Likert scale is commonly used in COS studies and recommended by the Grading of Recommendations Assessment, Development and Evaluation (GRADE) Working Group.[7 23]

For peer review only - http://bmjopen.bmj.com/site/about/guidelines.xhtml

Score criteria for consensus

- *Consensus in* is when ≥70% of participants rated 7-9 and <15% rated 1-3 for an outcome.[7 21]
- *Consensus out* is when <50% of participants rated 7-9 for an outcome.[7 21]
- *No consensus* is for any other scores.

This will be applied to the aggregate scores for all participants stratified by stakeholder groups.

Pilot study

The survey will be piloted before the Delphi rounds to check face validity. It will also inform the time frame required for completion of each Delphi round.

1st Delphi

Participants will be sent a participant information sheet explaining the objectives of the COS study. Completion of the online survey assumes implied consent. Participants will be informed that they can withdraw their response from the study within one week of submitting the survey. Once the name and contact details are separated from the survey response, it will not be possible to withdraw their survey response.

At the end of the survey, an open question will invite participants to suggest a maximum of two additional outcomes. If a new outcome is suggested by two or more participants, it will then be added to the 2nd Delphi round. Depending on how many new outcomes that will be presented, this criterion may be modified on a pragmatic basis.

2nd Delphi

Participants who responded to the 1st Delphi round will be invited to participate in the 2nd Delphi. A summary response from the 1st Delphi stratified by stakeholder groups will be presented for all outcomes.

3rd Delphi

Participants who responded to the 2nd Delphi round will be invited to participate in the 3rd Delphi. Outcomes that reached *consensus in* or *no consensus* in any of the stakeholder groups will be included as options in the 3rd Delphi survey. A summary response from the 2nd Delphi round, stratified by stakeholder groups will also be presented.

For peer review only - http://bmjopen.bmj.com/site/about/guidelines.xhtml

Stage 3: Consensus meeting

At the time of writing, the UK is undergoing social distancing due to the COVID-19 pandemic. In addition, our SAG patient representative has advised that travelling to meetings may not be convenient for mothers with childcare needs. Therefore, the consensus meeting will be conducted through a virtual platform online.

The consensus meeting panel will be purposefully selected from the SAG, PPI advisory group and Delphi survey respondents to ensure representation of a range of backgrounds. In the 3rd Delphi survey, participants will be asked about their willingness to attend the consensus meeting. For meaningful engagement in the consensus meeting, we will aim for 15-20 participants, with a minimum of five in each stakeholder group.[7 21 13]

An experienced facilitator will be the non-voting chair. Outcomes that have reached *consensus in* and *no consensus* in any of the stakeholder groups from the 3rd Delphi will be presented together with the summary scores. Discrepancy between stakeholder groups and equivocal outcomes will be discussed. Following that a final anonymous binary vote of yes /no will be conducted for each outcome. Outcomes that received ≥70% yes votes will be included in the final core outcome set.

For peer review only

Final COS

The final list will consist of a main COS that is applicable across all pregnancies and may consider disease specific subsets.

DISCUSSION

Strength

There is currently no COS for studies of pregnancy affected by multimorbidity. As multimorbidity covers a wide range of diseases, this presents a unique methodological challenge to the COS development. This study aims to adopt a pragmatic approach to make the task manageable whist still following the COS-STAD minimum standards. Inclusion of observational studies in generating the initial list of outcomes may detect rare but important clinical outcomes especially for offspring.[24]

The Delphi surveys and anonymous final vote in the consensus meeting will encourage participation of all stakeholders and avoid dominance of selected figures. As outlined in Figure 2, PPI will have a meaningful role throughout the COS development to ensure accessibility and relevance to patient stakeholder groups and that patient perspectives are represented in the governance of the COS development.[13]

Separating the COS into a main set generic to all pregnancies affected by multimorbidity and subsets specific to individual health conditions means the COS may be adapted for a wide range of research questions for researchers who may want to study specific combination or clusters of morbidities. In addition, to widen its applicability, the proposed COS will include both maternal and offspring outcomes. Finally, by creating this COS, we hope to encourage and facilitate urgently needed research into pregnancy affected by multimorbidity.

For peer review only - http://bmjopen.bmj.com/site/about/guidelines.xhtml

Limitation

Although a qualitative study is not planned, involvement of the SAG and PPI advisory group in reviewing the initial outcome list, and the opportunity for survey participants to suggest additional outcomes will ensure stakeholder's perspectives are considered.

For pragmatic reasons, the literature search will be limited to English studies. This may result in selection bias and limit the generalisability of the study findings to English speaking countries. Nevertheless, the findings will add substantial value to the current evidence base and will be readily available to researchers for future translation to other languages as required.

Due to the pragmatic literature search, the disease specific subset of COS may not be exhaustive for specific diseases. Nevertheless, we hope this will encourage more detailed research into relevant disease area.

DISSEMINATION

The final COS will be fed back to all stakeholders. Patient and public representatives will be encouraged and supported to share the difference they have made. With the guidance of the SAG and the PPI advisory group, a collaborative dissemination plan will be formulated. This will include submitting the findings for publication in a peer reviewed journal, dissemination at conferences and registering the study on the COMET database.

Authors' contributions: Our authors list includes PPI co-applicant NM and RP. All authors conceived the study; SIL led the development of the protocol and drafted the initial manuscript with contribution and supervision from KN, ST, MB, KAE; all authors contributed to the study design, critically reviewed and revised the protocol drafts. PPI co-applicant NM designed Figure 2. All authors agreed on the final draft manuscript for submission and are accountable for all aspects of the work.

Funding statement: This work was supported by Medical Research Council consolidator grant, grant number MR/V005243/1.

Competing interests: None declared.

Ethics: Appropriate ethical approval will be arranged with the University of Birmingham, Health Research Authority and National Health Service Research Ethics Committee as required.

Figure 1: Flowchart of COS development method

Figure 2: Description of patient and public involvement in the COS development

For peer review only - http://bmjopen.bmj.com/site/about/guidelines.xhtml

REFERENCES

1. NICE. Clinical knowledge summaries: Multimorbidity 2018. Available from: https://cks.nice.org.uk/multimorbidity#!backgroundSub (accessed 10 June 2020)
2. Gawron LM, Sanders JN, Sward K, et al. Multi-morbidity and Highly Effective Contraception in Reproductive-Age Women in the US Intermountain West: a Retrospective Cohort Study. *J Gen Intern Med* 2020;35(3):637-42. doi: 10.1007/s11606-019-05425-5
3. Graham W, Wood S, Byass P, et al. Diversity and divergence: the dynamic burden of poor maternal health. *Lancet* 2016;388(10056):2164-75. doi: 10.1016/s0140-6736(16)31533-1
4. Admon LK, Winkelman TNA, Heisler M, et al. Obstetric Outcomes and Delivery-Related Health Care Utilization and Costs Among Pregnant Women With Multiple Chronic Conditions. *Prev Chronic Dis* 2018;15:E21. doi: 10.5888/pcd15.170397
5. Beeson JG, Homer CSE, Morgan C, et al. Multiple morbidities in pregnancy: Time for research, innovation, and action. *PLoS Med* 2018;15(9):e1002665. doi: 10.1371/journal.pmed.1002665
6. Knight M, Tuffnell D, Kenyon S, et al. Saving Lives, Improving Mothers' Care - Surveillance of maternal deaths in the UK 2011-13 and lessons learned to inform maternity care from the UK and Ireland Confidential Enquiries into Maternal Deaths and Morbidity 2009-13. Oxford: National Perinatal Epidemiology Unit, University of Oxford 2015; 2015. https://www.npeu.ox.ac.uk/downloads/files/mbrrace-uk/reports/MBRRACE-UK%20Maternal%20Report%202015.pdf (accessed 1 June 2020)
7. Williamson PR, Altman DG, Bagley H, et al. The COMET Handbook: version 1.0. *Trials* 2017;18(Suppl 3):280-80. doi: 10.1186/s13063-017-1978-4
8. COMET initiative. Core Outcome Measures in Effectiveness Trials. http://www.comet-initiative.org/ (accessed 13 June 2020)
9. Kirkham JJ, Davis K, Altman DG, et al. Core Outcome Set-STAndards for Development: The COS-STAD recommendations. *PLoS Med* 2017;14(11):e1002447. doi: 10.1371/journal.pmed.1002447
10. Kirkham JJ, Gorst S, Altman DG, et al. Core Outcome Set–STAndards for Reporting: The COS-STAR Statement. *PLoS Med* 2016;13(10):e1002148. doi: 10.1371/journal.pmed.1002148
11. Williams K, Thomson D, Seto I, et al. Standard 6: Age Groups for Pediatric Trials. *Pediatrics* 2012;129(Supplement 3):S153-S60. doi: 10.1542/peds.2012-0055I
12. MuM-PreDiCT. Multimorbid Pregnancy: Determinants, Clusters, Consequences and Trajectories 2020. https://mumpredict.org/ (accessed 11 July 2020)
13. Moss N, Daru J, Lanz D, et al. Involving pregnant women, mothers and members of the public to improve the quality of women's health research. *BJOG* 2017;124(3):362-65. doi: 10.1111/1471-0528.14419
14. Al Wattar BH, Tamilselvan K, Khan R, et al. Development of a core outcome set for epilepsy in pregnancy (E-CORE): a national multi-stakeholder modified Delphi consensus study. *BJOG* 2017;124(4):661-67. doi: 10.1111/1471-0528.14430
15. Bain E, Pierides KL, Clifton VL, et al. Interventions for managing asthma in pregnancy. *Cochrane Database Syst Rev* 2014;2014(10):CD010660-CD60. doi: 10.1002/14651858.CD010660.pub2
16. Wang F, Lu JD, Zhu Y, et al. Renal Outcomes of Pregnant Patients with Immunoglobulin A Nephropathy: A Systematic Review and Meta-Analysis. *Am J Nephrol* 2019;49(3):214-24. doi: 10.1159/000496410
17. Dickinson F, McCauley M, Smith H, et al. Patient reported outcome measures for use in pregnancy and childbirth: a systematic review. *BMC Pregnancy Childbirth* 2019;19(1):155-55. doi: 10.1186/s12884-019-2318-3
18. Getahun D, Ananth CV, Oyelese Y, et al. Acute and chronic respiratory diseases in pregnancy: associations with spontaneous premature rupture of membranes. *J Matern Fetal Neonatal Med* 2007;20(9):669-75. doi: 10.1080/14767050701516063

For peer review only

19. Gargon E, Gorst SL, Williamson PR. Choosing important health outcomes for comparative effectiveness research: 5th annual update to a systematic review of core outcome sets for research. *PLoS One* 2019;14(12):e0225980. doi: 10.1371/journal.pone.0225980
20. Gargon E, Gurung B, Medley N, et al. Choosing important health outcomes for comparative effectiveness research: a systematic review. *PloS One* 2014;9(6):e99111-e11. doi: 10.1371/journal.pone.0099111
21. Egan AM, Dunne FP, Biesty LM, et al. Gestational diabetes prevention and treatment: a protocol for developing core outcome sets. *BMJ Open* 2019;9(11):e030574-e74. doi: 10.1136/bmjopen-2019-030574
22. Custer R, Scarcella J, Stewart B. The Modified Delphi Technique - A Rotational Modification. *Journal of Career and Technical Education* 1999;15(2):1-10.
23. GRADE. GRADE Handbook: 3. Selecting and rating the importance of outcomes 2013. https://gdt.gradepro.org/app/handbook/handbook.html (accessed 14 June 2020)
24. Williamson PR, Altman DG, Blazeby JM, et al. Developing core outcome sets for clinical trials: issues to consider. *Trials* 2012;13(1):132. doi: 10.1186/1745-6215-13-132

For peer review only - http://bmjopen.bmj.com/site/about/guidelines.xhtml

Figure 1: Flowchart of COS development method

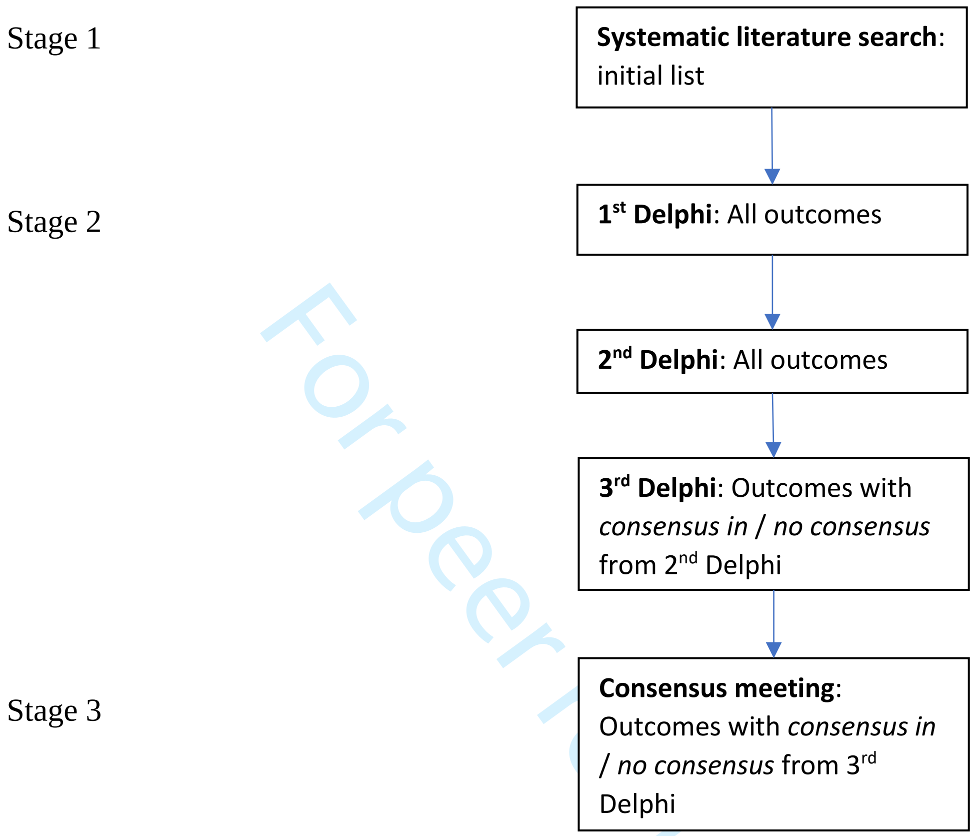

NB: For the 2nd, 3rd Delphi surveys and the consensus meeting, an aggregate score from the previous round, stratified by stakeholder groups, will be presented.

For peer review only

Figure 2: Description of patient and public involvement in the core outcome set development

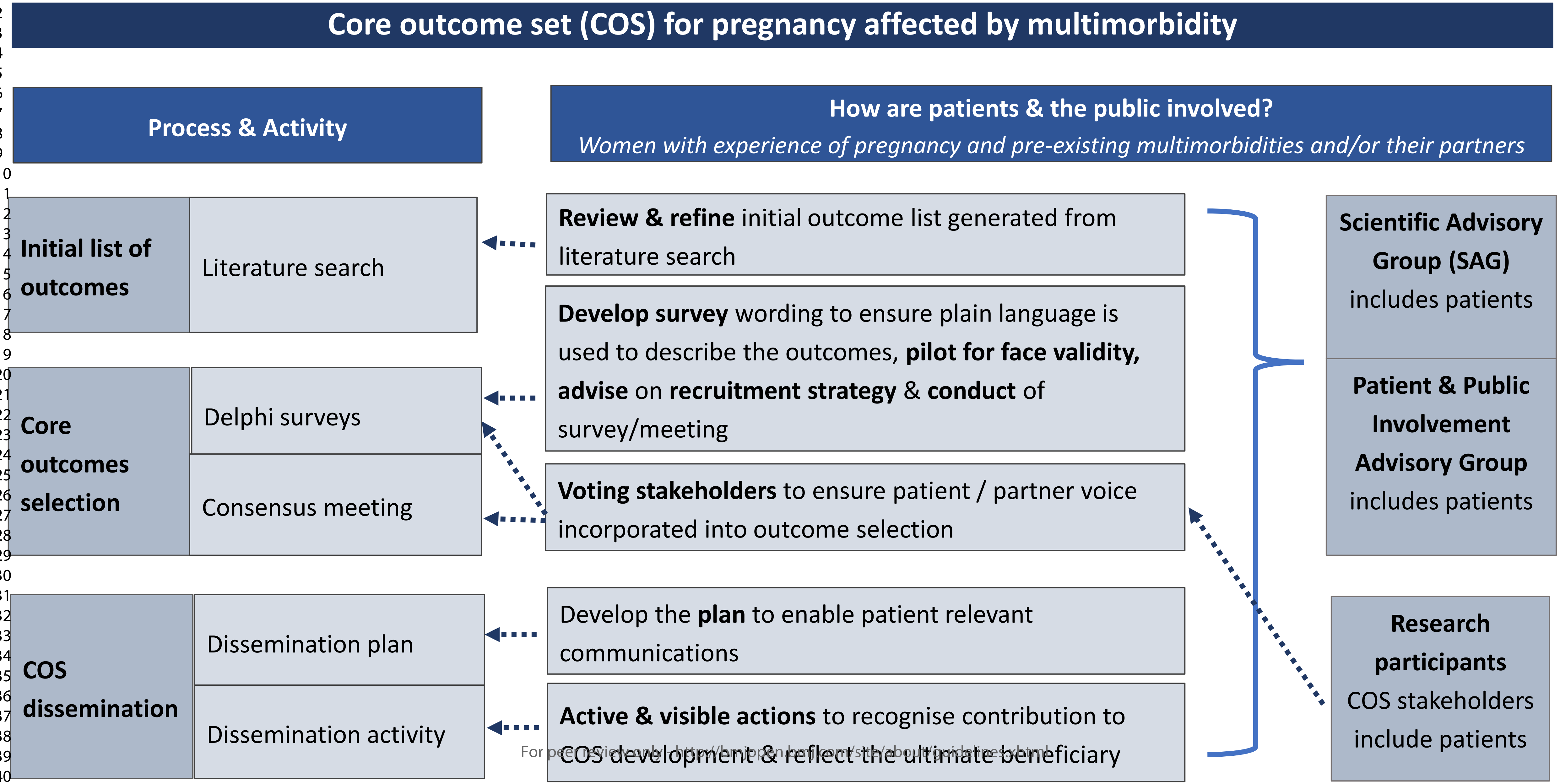

For peer review only - http://bmjopen.bmj.com/site/about/guidelines.xhtml

APPENDIX

Example literature search strategies for each level of the planned hierarchical search

Core outcome set (COS)

Database: COMET

Exposure: Epilepsy

Search strategy:

1. Epilepsy in pregnancy
2. Both published and unpublished

Systematic review

Database: Cochrane library

Exposure: Chronic kidney disease

Search strategy:

#1 chronic kidney disease

#2 chronic renal disease

#3 MeSH descriptor: [Renal Insufficiency, Chronic] explode all trees

#4 pregnancy

#5 MeSH descriptor: [Pregnancy] explode all trees

#6 #1 or #2 or #3

#7 #4 or #5

#8 #6 and #7

Systematic review

Database: Ovid MEDLINE(R) and In-Process & Other Non-Indexed Citations

Exposure: Chronic kidney disease

Search strategy:

1. exp Pregnancy/ or pregnancy.mp.
2. systematic review.mp. or exp "Systematic Review"/
3. chronic renal disease.mp.
4. chronic kidney disease.mp. or exp Renal Insufficiency, Chronic/

For peer review only - http://bmjopen.bmj.com/site/about/guidelines.xhtml

5. 3 or 4
6. 1 and 2 and 5

Patient reported outcome measures (PROM)

Database: Ovid MEDLINE(R) and In-Process & Other Non-Indexed Citations

Exposure: Chronic obstructive pulmonary disease

Search strategy:

1. exp Pregnancy/ or pregnancy.mp.
2. chronic obstructive pulmonary disease.mp. or exp Pulmonary Disease, Chronic Obstructive/
3. chronic obstructive respiratory disease.mp.
4. chronic obstructive lung disease.mp.
5. chronic obstructive airway disease.mp.
6. emphysema.mp. or exp Emphysema/ or exp Pulmonary Emphysema/
7. chronic bronchitis.mp. or exp Bronchitis, Chronic/
8. or/2-7
9. patient reported outcome measures.mp. or exp Patient Reported Outcome Measures/
10. 1 and 8 and 9

For peer review only

Primary studies

Database: Ovid MEDLINE(R) and In-Process & Other Non-Indexed Citations

Exposure: Chronic obstructive pulmonary disease

Search strategy:

1. exp Pregnancy/ or pregnancy.mp.
2. chronic obstructive pulmonary disease.mp. or exp Pulmonary Disease, Chronic Obstructive/
3. chronic obstructive respiratory disease.mp
4. chronic obstructive lung disease.mp
5. chronic obstructive airway disease.mp

For peer review only - http://bmjopen.bmj.com/site/about/guidelines.xhtml

6. emphysema.mp. or exp Emphysema/ or exp Pulmonary Emphysema/

7. chronic bronchitis.mp. or exp Bronchitis, Chronic/

8. 2 or 3 or 4 or 5 or 6 or 7

9. 1 and 8

For peer review only

For peer review only - http://bmjopen.bmj.com/site/about/guidelines.xhtml

BMJ Open

Protocol for the development of a core outcome set for studies of pregnant women with pre-existing multimorbidity

Journal:	*BMJ Open*
Manuscript ID	bmjopen-2020-044919.R1
Article Type:	Protocol
Date Submitted by the Author:	27-Apr-2021
Complete List of Authors:	Lee, Siang Ing; University of Birmingham Institute of Applied Health Research,<br>Eastwood, Kelly-Ann; Queen's University Belfast, Centre for Public Health; University Hospitals Bristol and Weston NHS Foundation Trust, St Michael's Hospital<br>Moss, Ngawai; Patient and Public Representative<br>Azcoaga-Lorenzo, Amaya; University of St Andrews, Division of Population and Behavioural Sciences, School of Medicine<br>Subramanian, Anuradha; University of Birmingham Institute of Applied Health Research<br>Anand, Astha; University of Birmingham Institute of Applied Health Research<br>Taylor, Beck; University of Birmingham Institute of Applied Health Research<br>Nelson-Piercy, Catherine; Guy's and Saint Thomas' NHS Foundation Trust<br>Yau, Christopher; The University of Manchester, Division of Informatics, Imaging and Data Sciences, Faculty of Biology Medicine and Health<br>McCowan, Colin ; University of St Andrews, School of Medicine<br>OReilly, Dermot; Queen's University Belfast, Epidemiology and Public Health<br>Hope, Holly; The University of Manchester Faculty of Biology Medicine and Health, Centre for Women's Mental Health, Division of Psychology and Mental Health, School of Health Sciences<br>Kennedy, Jonathan; Swansea University College of Medicine<br>Abel, Kathryn ; The University of Manchester Faculty of Biology Medicine and Health, Centre for Women's Mental Health, Division of Psychology and Mental Health; Greater Manchester Mental Health NHS Foundation Trust<br>Locock, Louise; University of Aberdeen, Health Service Research<br>Brocklehurst, Peter; University of Birmingham Institute of Applied Health Research<br>Plachcinski, Rachel; Patient and Public Representative<br>Brophy, Sinead; Swansea University College of Medicine<br>Agrawal, Utkarsh; University of St Andrews, School of Medicine<br>Thangaratinam, Shakila; University of Birmingham, Institute of Metabolism and Systems Research<br>Nirantharakumar, Krishnarajah; University of Birmingham Institute of Applied Health Research<br>Black, Mairead; University of Aberdeen, School of Medicine, Medical Science and Nutrition

**Primary Subject Heading**:	Obstetrics and gynaecology
Secondary Subject Heading:	Research methods
Keywords:	Maternal medicine < OBSTETRICS, PUBLIC HEALTH, PREVENTIVE MEDICINE

SCHOLARONE™ Manuscripts

I, the Submitting Author has the right to grant and does grant on behalf of all authors of the Work (as defined in the below author licence), an exclusive licence and/or a non-exclusive licence for contributions from authors who are: i) UK Crown employees; ii) where BMJ has agreed a CC-BY licence shall apply, and/or iii) in accordance with the terms applicable for US Federal Government officers or employees acting as part of their official duties; on a worldwide, perpetual, irrevocable, royalty-free basis to BMJ Publishing Group Ltd ("BMJ") its licensees and where the relevant Journal is co-owned by BMJ to the co-owners of the Journal, to publish the Work in this journal and any other BMJ products and to exploit all rights, as set out in our licence.

The Submitting Author accepts and understands that any supply made under these terms is made by BMJ to the Submitting Author unless you are acting as an employee on behalf of your employer or a postgraduate student of an affiliated institution which is paying any applicable article publishing charge ("APC") for Open Access articles. Where the Submitting Author wishes to make the Work available on an Open Access basis (and intends to pay the relevant APC), the terms of reuse of such Open Access shall be governed by a Creative Commons licence – details of these licences and which Creative Commons licence will apply to this Work are set out in our licence referred to above.

Other than as permitted in any relevant BMJ Author's Self Archiving Policies, I confirm this Work has not been accepted for publication elsewhere, is not being considered for publication elsewhere and does not duplicate material already published. I confirm all authors consent to publication of this Work and authorise the granting of this licence.

For peer review only

Title: Protocol for the development of a core outcome set for studies of pregnant women with pre-existing multimorbidity

Authors: Siang Ing Lee,[1] Kelly-Ann Eastwood,*[2, 3] Ngawai Moss,*[4] Amaya Azcoaga-Lorenzo,[5] Anuradhaa Subramanian,[1] Astha Anand,[1] Beck Taylor,[1] Catherine Nelson-Piercy,[6] Christopher Yau,[7] Colin McCowan,[5] Dermot O'Reilly,[2] Holly Hope,[8] Jonathan I Kennedy,[9] Kathryn M Abel,[8, 10] Louise Locock,[11] Peter Brocklehurst,[1] Rachel Plachcinski,[4] Sinead Brophy,[9] Utkarsh Agrawal,[5] Shakila Thangaratinam,*[12] Krishnarajah Nirantharakumar,*[1] Mairead Black*[11]

* indicates equal contribution

Affiliation:

1. Institute of Applied Health Research, University of Birmingham, Birmingham, UK
2. Centre for Public Health, Queen's University of Belfast
3. St Michael's Hospital, University Hospitals Bristol and Weston NHS Foundation Trust, Bristol, UK
4. Patient and public representative
5. School of Medicine, University of St Andrews, UK
6. Guy's and St. Thomas' NHS Foundation Trust
7. Division of Informatics, Imaging and Data Sciences, Faculty of Biology Medicine and Health, The University of Manchester
8. Centre for Women's Mental Health, Division of Psychology and Mental Health, School of Health Sciences, Faculty of Biology Medicine & Health, The University of Manchester, UK
9. Data Science, Medical School, Swansea University, UK
10. Greater Manchester Mental Health NHS Foundation Trust, Manchester
11. School of Medicine, Medical Science and Nutrition, University of Aberdeen, UK
12. Institute of Metabolism and Systems Research, University of Birmingham, Birmingham, UK

Authors ORCID: Siang Ing Lee 0000-0002-2332-5452, Kelly-Ann Eastwood 0000-0003-3689-0490, Ngawai Moss 0000-0001-9369-5072, Amaya Azcoaga-Lorenzo 0000-0003-3307-878X, Anuradhaa Subramanian 0000-0001-8875-7363, Astha Anand 0000-0003-0494-321X, Beck Taylor 0000-0002-3559-7922, Catherine Nelson-Piercy 0000-0001-9311-1196, Christopher Yau 0000-0001-7615-8523, Colin McCowan 0000-0002-9466-833X, Dermot O'Reilly 0000-0002-9181-0652, Holly Hope 0000-0002-4834-6719, Jonathan I Kennedy 0000-0002-1122-6502, Kathryn M Abel 0000-0003-3538-8896, Louise Locock 0000-0002-8109-1930, Peter Brocklehurst 0000-0002-9950-6751, Rachel Plachcinski 0000-0001-9908-0773, Sinead Brophy 0000-0001-7417-2858, Utkarsh Agrawal 0000-0001-5181-6120, Shakila Thangaratinam 0000-0002-4254-460X, Krishnarajah Nirantharakumar 0000-0002-6816-1279, Mairead Black 0000-0002-6841-8601

Corresponding author: Krishnarajah Nirantharakumar, K.Nirantharan@bham.ac.uk, IOEM Building, University of Birmingham, Edgbaston, Birmingham, B15 2TT, UK
Word count: 2798
Figures: 2
Tables: 2
Appendix: 1
Keywords: multimorbidity, multiple long-term conditions, multiple chronic conditions, pregnancy, obstetrics, maternity, core outcome set

For peer review only - http://bmjopen.bmj.com/site/about/guidelines.xhtml

ABSTRACT

Introduction

Increasingly more pregnant women are living with pre-existing multimorbidity (≥2 long-term physical or mental health conditions). This may adversely affect maternal and offspring outcomes. This study aims to develop a COS for maternal and offspring outcomes in pregnant women with pre-existing multimorbidity. It is intended for use in observational and interventional studies in all pregnancy settings.

Methods and analysis

We propose a four stage study design: 1) systematic literature search, 2) focus groups, 3) Delphi surveys, and 4) consensus group meeting. The study will be conducted from June 2021 – August 2022.

First, an initial list of outcomes will be identified through a systematic literature search of reported outcomes in studies of pregnant women with multimorbidity. We will search the Cochrane library, Medline, EMBASE and CINAHL. This will be supplemented with relevant outcomes from published COS for pregnancies and childbirth in general, and multimorbidity. Second, focus groups will be conducted amongst 1) women with lived experience of managing pre-existing multimorbidity in pregnancy (and/or their partners), and 2) their health/social care professionals to identify outcomes important to them.

Third, these initial lists of outcomes will be prioritised through a three-round online Delphi survey using predefined score criteria for consensus. Participants will be invited to suggest additional outcomes that were not included in the initial list. Finally, a consensus meeting using the nominal group technique will be held to agree on the final COS. The stakeholders will include 1) women (and/or their partners) with lived experience of managing multimorbidity in pregnancy, 2) health/social care professionals involved in their care, and 3) researchers in this field.

Ethics and dissemination

This study has been approved by the University of Birmingham's Ethical Review Committee. The final COS will be disseminated through peer-reviewed publication and conferences and to all stakeholders.

294 words

Strengths and limitations of this study

- Core outcome set (COS) development in accordance to the COS standards for development (COS-STAD)
- Extensive patient, public and stakeholder involvement at each stage
- Pragmatic design to make the COS development feasible in the context of multimorbidity
- The applicability of the COS may be limited to high income countries
- Responder bias may influence the types of outcomes included in the final COS

For peer review only

BACKGROUND

Multimorbidity is a state of having two or more long-term physical or mental health conditions.[1] Despite an increase in multimorbidity within the general population,[2] there is sparse literature for pregnant women with multimorbidity. Studies in the USA have reported that between 0.8% to 13.9% of hospital births were from women with multiple chronic conditions.[3 4] Using a list of 79 chronic conditions, our preliminary study found that one in four pregnant women in the UK had active multimorbidity at conception.[5]

Studies have shown that multimorbidity is associated with increased risk of adverse obstetric outcomes (e.g. preterm birth) and severe maternal morbidities as a consequence of childbirth (e.g. hysterectomy, eclampsia).[3 4] The 2020 UK national maternal mortality review reported that 90% of women who died within a year of pregnancy had multiple health and social problems.[6] The leading direct cause of maternal death included thrombosis, thromboembolism and maternal suicide; leading indirect cause of death included cardiac diseases, epilepsy and stroke.[6] In addition to acute complications (e.g. eclampsia) and chronic complications (progression from gestational diabetes to type II diabetes) for the mother, evidence suggests that pre-existing maternal morbidities and medications taken for these morbidities can lead to offspring complications such as neurodevelopmental disorders and congenital anomalies.[4 7-10] Current observational evidence and interventions focus on single morbidities. There is an urgent need for further understanding of the consequence of pre-existing maternal multimorbidity and development of interventions to improve maternity care for these women.[11 12]

To facilitate future research studies, a core outcome set (COS) is required. This will standardise the outcomes being reported, allow for evidence synthesis, and ensure outcomes important to women, their families, carers and health and social care professionals are captured.[13] The importance of COS in women's health is endorsed by the Core Outcomes in Women's Health (CROWN) initiative.[14] The Core Outcome Measures in Effectiveness Trial (COMET) initiative collates resources for COS development and maintains a COS database.[15]

A recent scoping review identified 26 COSs relevant to maternity service users, of which three were related to pre-existing maternal morbidities in pregnancy (diabetes, epilepsy, infertility).[16] A search for COS in pregnancy on the COMET database further identified two published COS (depression, rheumatological conditions) and three in progress (cardiac disease, venous thromboembolism and immune thrombocytopenia).[15] There is currently no COS for multimorbidity in pregnancy. We propose a pragmatic study design to develop a COS for observational and interventional studies, for pregnant women with pre-existing multimorbidity, covering obstetrics, maternal and offspring outcomes.

METHODS

This study is designed in accordance with the COS standards for development (COS-STAD) recommendations and the protocol follows the COS-STAP statement (Appendix 1); study findings will be reported following the COS standards for reporting (COS-STAR).[17-19] The planned start and end dates for the study are June 2021 and August 2022, respectively. The study is registered on the COMET database.[20]

For peer review only - http://bmjopen.bmj.com/site/about/guidelines.xhtml

The study will consist of four stages: 1) systematic literature search for reported outcomes for mother and child in studies of pregnant women with multimorbidity; 2) focus groups of women with lived experience of managing pre-existing multimorbidity in pregnancy and/or their partners, and their health/social care professionals; 3) Delphi surveys amongst stakeholders to prioritise the core outcomes; and 4) a consensus meeting to agree on the final COS (Figure 1).

Scope of the COS

The population is pregnant women; the exposure is pre-existing multimorbidity, defined as having two or more long-term physical or mental health conditions at conception.[1] This does not include pregnancy related morbidities (e.g. gestational diabetes) which will be considered as pregnancy outcomes. The morbidities do not have to be independent of each other. For instance, if a morbidity is a consequence of another morbidity (e.g. diabetic eye disease and diabetes), these will be classed as two separate morbidities. The COS will be applicable principally to observational studies but will also inform interventional studies for pregnancy in all settings.

Maternal outcomes will include the antenatal, intrapartum and post-partum period. Offspring outcomes will include the neonatal (first one month), infant (first one year), pre-pubertal (two to 11 years old), pubertal period (12-18 years old) and adulthood.[21] We have included outcomes across the lifespan of the offspring to inform observational studies that take a life-course approach.[22] Evidence is emerging that pre-existing maternal morbidities can impact on offspring long-term health in early adulthood.[23] Pregnancy outcomes in the rest of this protocol will refer to both maternal and offspring outcomes.

Patient and public involvement (PPI)

This protocol has been shaped by extensive PPI. PPI for this study will be three-tiered: (1) patient representatives in the scientific advisory group (SAG), (2) PPI advisory group and (3) patient and public stakeholders as research participants.

The SAG consists of clinicians (specialists in maternal and fetal medicine, obstetrics, perinatal mental health, general practice and public health), researchers and women representatives collaborating on a larger project studying pregnant women with multimorbidity (MuM-PreDiCT).[24] NM, a women representative from the SAG has advised on the study design, co-authored this protocol and created Figure 2 that illustrates the PPI in the COS development.[25]

Stage 1: Systematic literature search

A pragmatic approach to identifying a list of initial outcomes will be adopted given the wide range of potential multimorbidities. We will first identify outcomes from published COS for pregnancy and childbirth and published COS for multimorbidity from the COMET database.[26-29] We will then conduct a systematic literature search for reported outcomes in published studies of pregnant women with multimorbidity.

For peer review only - http://bmjopen.bmj.com/site/about/guidelines.xhtml

Search strategy

The following databases will be searched: Cochrane library, Medline, EMBASE and CINAHL. Relevant key search terms will include pregnancy (population and maternal outcomes), multimorbidity (exposure) and offspring (offspring outcomes) derived from previous literature.[28 30 31]

Study selection and data extraction

The inclusion criteria are: systematic reviews, interventional studies, observational studies, qualitative studies and patient reported outcome measures (PROM) studies; studies reporting pregnancy, maternal and offspring outcomes; and studies of pregnant women with multimorbidity. The exclusion criteria are: ongoing studies with no published outcomes, editorials, commentaries, narrative reviews, case reports, case series, diagnostic accuracy studies, laboratory studies and animal studies. No time or language limits will be applied. Full text screening will be conducted by two independent reviewers.

Two reviewers will extract the following data from included studies: author, year of publication, study design, PROM domains, types of outcomes, definition of and measurement tools for the outcomes. Any discrepancy between the two independent reviewers for study selection and data extraction will be resolved with a third reviewer.

Stage 2: Focus groups

Outcomes identified in the published literature may represent outcomes considered as important to researchers.[13] Therefore, focus groups will be conducted to ensure the capture of outcomes considered as important to women with lived experience of managing pre-existing multimorbidity in pregnancy and/or their carers/partners (two focus groups), and health/social care professionals involved in their care (one focus group). The synergistic discussion in focus groups will allow participants to consider outcomes which are important to others and stimulate in-depth discussions.[32]

We will aim to include 6-8 participants per focus group. Sampling will be purposive and guided by the sampling matrix to provide a broad representation of stakeholders and characteristics (Table 1). Recruitment channels are listed in Table 2. Involvement of the under-served population will be guided by our PPI advisory group and the MuM-PreDiCT group's strategy for diverse representation.[5]

Based on the advice of our PPI advisory group, the focus groups will be held virtually. Participants will be sent participant information sheets in advance of the meeting and consent will be taken 24 hours later either in electronic form or verbally. The focus group will last for 90 minutes or until no further new ideas are forthcoming. A topic guide will be developed based on previous literature, and with the guidance of qualitative experts and patient representatives in the SAG and our PPI advisory group.[33 34] The focus group will be facilitated by a researcher with qualitative methodology training. The focus group discussion will be recorded using the virtual meeting platform, the recordings will be transcribed and imported to NVivo. Data analysis will be inductive, following a structured, multistage approach to thematic analysis.[35]

For peer review only - http://bmjopen.bmj.com/site/about/guidelines.xhtml

Table 1: Sampling matrix for the focus groups, Delphi surveys and consensus meeting

Characteristics	Target / minimum numbers		
	**Focus groups**	**Delphi surveys[36]**	**Consensus meeting**
**1) Women with lived experience of managing pre-existing multimorbidity (2+ long-term conditions) in pregnancy**	12-16	50	5
Physical health conditions	6	8-10	1
Mental health conditions	3-6	8-10	1
Ethnic minority	3-6	8-10	2
Socioeconomically disadvantaged/ marginalised groups (e.g. homeless, refugee, asylum seeker, drug and alcohol service users, disabled, victims of domestic abuse)[6]	3-6	8-10	1
**2) Health / social care professionals**	6-8	50	5
Obstetric medicine / maternal medicine	1-2	8-10	1
Obstetric	1-2	8-10	1
Midwifery / antenatal practitioner	1-2	8-10	1
Perinatal mental health	1-2	8-10	1
Other: e.g. primary care, public health, neonatologist, paediatrician, health visitor, commissioner, maternity service provider, social worker, drug and alcohol service provider, maternity advocate /educator	2	8-10	1
**3) Researchers** Academics, triallist, journal editors (as future implementers)	-	5-10	2

NB: *Target/minimum numbers are estimates. Due to the overlap of characteristics between participants (e.g. physical and mental health conditions, health/social care professionals and researchers) we will continuously review the characteristics of participants so that we can identify any under-represented groups and target recruitment efforts in these areas.

For peer review only - http://bmjopen.bmj.com/site/about/guidelines.xhtml

Initial list of outcomes

The initial list of outcomes generated from stages 1 and 2 will be reviewed and refined by the SAG and PPI advisory group to combine outcomes that are clinically and pathophysiologically similar to avoid redundancy.[13 37] Pregnancy outcomes will be categorised by: (1) maternal or offspring outcomes, and (2) by an established taxonomy of outcomes (mortality/survival, physiological/clinical, life impact/functioning, resource use and adverse events/effects).[38]

Stage 3: Delphi surveys

The Delphi technique collates stakeholder opinions using sequential surveys. The response is summarised and fed back to stakeholders anonymously in subsequent rounds. Stakeholders consider the collective views before re-rating the outcomes. This provides a mechanism to reconcile different opinions to reach a consensus.[13] This study will employ a three round Delphi survey which is generally sufficient to reach consensus (Figure 1).[39] Participants will have the opportunity to suggest additional outcomes that were not included in the initial list.

The surveys will be hosted on a secure platform online. The three groups of stakeholders that will be invited to participate and the recruitment channels are outlined in Table 2. There is no recommended sample size for Delphi surveys; instead of basing the sample size on statistical power, this is often a pragmatic choice.[13] Previous obstetric COS has achieved sample size of around 20-40 for patients and 50-100 for health care professionals.[37 40-42] To reach the target sample size, snowballing recruitment will be encouraged. To check for representation, the survey will ask for participant characteristics including types of long-term conditions constituting multimorbidity, age, ethnicity, education level and socioeconomic status (patient representatives, as outlined in Table 1), specialty and job roles (health care professionals and researchers). Participant's name and email contact will be included to avoid duplicate entry, for sending up to two personalised reminders (one week apart) and following up on incomplete response. This information will be kept securely, confidentially and separate from the survey responses.

For peer review only

For peer review only - http://bmjopen.bmj.com/site/about/guidelines.xhtml

Table 2: Stakeholders and recruitment channels

Stakeholder group	Potential recruitment channels[43 44]
**1) Patient representatives**<br>Women with lived experience of managing pre-existing multimorbidity (two or more long-term physical or mental health conditions) in pregnancy and/or their partners/carers	• Service user associations/groups: e.g. Maternity Voice Partnership<br>• Parent support networks: e.g. National Childbirth Trust<br>• Community groups: local maternity groups, baby/toddler groups, local authority baby class, nursery, health visitor society, faith group, baby groups by church<br>• Social media: Facebook, Twitter, Instagram, Linkedin<br>• Parent oriented social media: home-schooling, weaning, budget family menu sites, breastfeeding, outdoor activities for family, local outdoor groups, Mumsnet, Gingerbread (single parents)<br>• Patient support groups/charities for specific conditions: Tommy's, Epilepsy Action, Association of Medical Research UK member charities, National Council for Voluntary Organisations<br>• Royal Colleges women's networks: Royal College of Obstetrics and Gynaecology Women's Voices Involvement Panel, Royal College of Midwifery Maternity Voices Network<br>• Victim of domestic abuse: Refuge, Women's Aid, WE:ARE (Women's Empowerment and Recovery Educators)<br>• Disabled: Disabled Parents Network, disabled parents Facebook groups<br>• Drug and alcohol: Drug and Alcohol Abuse Support for Women<br>• Refugee: Refugee Council, Refugee Survival Trust<br>• LGBT: LGBT Mummies Tribe, Stonewall, Facebook groups for transgender men or lesbian women experiencing pregnancy
**2) Health / social care professionals**<br>Any health/ social care professionals involved in providing multidisciplinary team care for pregnant women: e.g. obstetric physicians, obstetricians, physicians, paediatricians, neonatologists, psychiatrists, primary care clinicians, public health professionals, clinicians of established joint antenatal clinics, perinatal mental health team, drug and alcohol services, social services, midwives, health visitors, dieticians, policy makers, commissioners.	- Personal, professional and clinical network of the researchers<br>- Royal colleges<br>- Societies (e.g. McDonald Obstetric Medicine Society, European Board and College of Obstetrics and Gynaecology)<br>- Maternity charities (e.g. Ammalife, Elly)<br>- Social media for professional groups (e.g. Twitter, Facebook).
**3) Researchers**<br>Academics, triallist, journal editors (as future implementers)	The SAG's personal network, social media (Twitter), the COMET and Core Outcomes in Women's Health (CROWN) network, the Cochrane Pregnancy and Childbirth group, peer-reviewed journals of obstetric medicine and obstetrics

For peer review only - http://bmjopen.bmj.com/site/about/guidelines.xhtml

Care will be taken in explaining the concept of COS to lay participants, using supporting materials from the COMET website.[15] The wording of the survey will be developed using appropriate language commonly used by representatives in the focus groups. The SAG and PPI advisory group will also ensure plain language is used to describe the outcomes of interest. Outcomes will be presented in alphabetical order to avoid any response effects related to the order of survey items.[13 45]

Each outcome will be rated on a 9-point Likert scale: 1-3 (not important), 4-6 (important but not critical) and 7-9 (critically important). An '*unable to score'* option will be provided to allow for participants who may not have the expertise to score certain outcomes.[13] The 9-point Likert scale is commonly used in COS studies and recommended by the Grading of Recommendations Assessment, Development and Evaluation (GRADE) Working Group.[13 46]

Score criteria for consensus[47]

- *Consensus in* is when ≥70% of all participants rated 7-9 (critically important) for an outcome.
- *Consensus out* is when ≥70% of all participants rated 1-3 (not important) for an outcome.
- *No consensus* is for any other scores.
- *For further discussion* is when: (1) ≥70% of all participants rated 4-6 (important but not critical) for an outcome, or (2) when ≥70% of patient representatives have rated 7-9 for an outcome but *consensus in* is not reached.

Pilot study

The survey will be piloted before the Delphi rounds to check face validity. It will also inform the time frame required for completion of each Delphi round.

1st Delphi

Participants will be sent a participant information sheet explaining the objectives of the COS study. Completion of the online survey assumes implied consent. Participants will be informed that they can withdraw their response from the study within one week of submitting the survey. Once the name and contact details are separated from the survey response, it will not be possible to withdraw their survey response.

At the end of the survey, an open question will invite participants to suggest a maximum of two additional outcomes. If a new outcome is suggested by two or more participants, it will then be added to the 2nd Delphi round. Depending on how many new outcomes that will be presented, this criterion may be modified on a pragmatic basis.

2nd Delphi

Participants who responded to the 1st Delphi round will be invited to participate in the 2nd Delphi. A summary response from the 1st Delphi stratified by stakeholder groups will be presented for all outcomes.

3rd Delphi

Participants who responded to the 2nd Delphi round will be invited to participate in the 3rd Delphi. Outcomes that reached *no consensus* will be included as options in the 3rd Delphi

For peer review only - http://bmjopen.bmj.com/site/about/guidelines.xhtml

survey. A summary response from the 2nd Delphi round, stratified by stakeholder groups will also be presented. Attrition rate will be calculated for each subsequent rounds.

Stage 4: Consensus meeting

At the time of writing, the UK is undergoing social distancing due to the COVID-19 pandemic. In addition, our SAG patient representative has advised that travelling to meetings may not be convenient for mothers with childcare needs. Therefore, the consensus meeting will be conducted through a virtual platform online.

The consensus meeting panel will be purposefully selected from the SAG, PPI advisory group and Delphi survey respondents to ensure representation of a range of backgrounds. In the 3rd Delphi survey, participants will be asked about their willingness to attend the consensus meeting. For meaningful engagement in the consensus meeting, we will aim for 10-15 participants.[13 25 45]

An experienced facilitator will be the non-voting chair. Summary scores stratified by stakeholder groups will be presented for outcomes that met the *'for further discussion'* criteria. Nominal group technique will be used to discuss these outcomes.[47 48] Participants will be asked to contemplate independently whether these outcomes should be included. Each participant will be invited to voice their reasoning in turn using a round-robin format to avoid domination of the discussion by selected few. This will be followed by an open discussion, after which a final anonymous binary vote of yes /no will be conducted for each of these outcomes. Outcomes that received ≥70% yes votes will be included in the final COS.

DISCUSSION

The proposed COS will be applicable for observational and interventional studies for pregnant women with pre-existing multimorbidity. Further interventional studies are urgently needed to tackle multimorbidity in pregnancy and reduce the associated adverse outcomes. It is therefore important to have a predefined COS to inform future research studies to enable valid comparisons between study findings.

Strength

There is currently no COS for studies of pregnant women with multimorbidity. As multimorbidity covers a wide range of diseases, this presents a unique methodological challenge to the COS development. This study aims to adopt a pragmatic approach to make the task manageable whilst still following the COS-STAD minimum standards. Inclusion of observational studies in generating the initial list of outcomes may detect rare but important clinical outcomes especially for offspring.[49]

The Delphi surveys, nominal group technique and anonymous final vote in the consensus meeting will encourage participation of all stakeholders and avoid dominance of selected figures. As outlined in Figure 2, PPI will have a meaningful role throughout the COS

For peer review only - http://bmjopen.bmj.com/site/about/guidelines.xhtml

development to ensure accessibility and relevance to patient stakeholder groups and that patient perspectives are represented in the governance of the COS development.[25]

To widen its applicability, the proposed COS will include both maternal and offspring outcomes and will include outcomes that are common to all pregnant women with multimorbidity. Finally, by creating this COS, we hope to encourage and facilitate urgently needed research for pregnant women with multimorbidity.

Limitation

The focus groups, Delphi survey and consensus meeting will be conducted in English. Although efforts will be made to encourage international participation, this may limit the generalisability of the findings to high income countries. The use of online platforms may lead to under-representation of the digitally disadvantaged groups. Similarly, responder bias may influence the types of outcomes included in the final COS. To ensure representation of the socially disadvantaged / marginalised group and health/social care professionals with busy work schedules, our approach will be flexible and where necessary / preferred by the participants, we will offer the option of one-to-one interviews instead of focus groups.

As further epidemiological knowledge is gained in identifying common morbidity clusters in pregnant women, the COS may need to be updated to incorporate outcomes specific to these clusters.

DISSEMINATION

The final COS will be fed back to all stakeholders. Patient and public representatives will be encouraged and supported to share the difference they have made. With the guidance of the SAG and the PPI advisory group, a collaborative dissemination plan will be formulated. This will include submitting the findings for publication in a peer reviewed journal, dissemination at conferences and registering the study on the COMET database.

Authors' contributions: Our authors list includes PPI co-investigators NM and RP. SIL, KAE, NM, AAL, AS, AA, BT, CNP, CY, CM, DOR, HH, JIK, KMA, LL, PB, RP, SB, UA, ST, KN and MB conceived the study; SIL led the development of the protocol and drafted the initial manuscript with contribution and supervision from KN, ST, MB, KAE; SIL, KAE, NM, AAL, AS, AA, BT, CNP, CY, CM, DOR, HH, JIK, KMA, LL, PB, RP, SB, UA, ST, KN and MB contributed to the study design, critically reviewed and revised the protocol drafts. LL, BT, MB contributed to the qualitative element of the study design and, together with NM, RP, SB and KMA, advised on the recruitment channels; PPI co-investigator NM designed Figure 2. SIL, KAE, NM, AAL, AS, AA, BT, CNP, CY, CM, DOR, HH, JIK, KMA, LL, PB, RP, SB, UA, ST, KN and MB agreed on the final draft manuscript for submission and are accountable for all aspects of the work.

Funding statement: This work was supported by Medical Research Council consolidator grant, grant number MR/V005243/1.

For peer review only - http://bmjopen.bmj.com/site/about/guidelines.xhtml

Competing interests: None declared.

Ethics: This project has been reviewed and approved by the Science, Technology, Engineering and Mathematics Ethical Review Committee at the University of Birmingham (reference: ERN_20-1264A). Health Research Authority (HRA) has advised that this project does not require HRA and HCRW approval or NHS/HSC R&D permissions as it is a service evaluation/improvement project.

Figure 1: Flowchart of COS development method

Figure 2: Description of patient and public involvement in the COS development

For peer review only

REFERENCES

1. The Academy of Medical Sciences. Multimorbidity: a prority for global health research 2018. Available: https://acmedsci.ac.uk/file-download/99630838 [Accessed 22 March 2021].
2. Whitty CJM, MacEwen C, Goddard A, et al. Rising to the challenge of multimorbidity. *BMJ* 2020;368:l6964. doi: 10.1136/bmj.l6964
3. Admon LK, Winkelman TNA, Heisler M, et al. Obstetric Outcomes and Delivery-Related Health Care Utilization and Costs Among Pregnant Women With Multiple Chronic Conditions. *Preventing chronic disease* 2018;15:E21. doi: 10.5888/pcd15.170397
4. Brown CC, Adams CE, George KE, et al. Associations Between Comorbidities and Severe Maternal Morbidity. *Obstetrics and gynecology* 2020;136(5):892-901. doi: 10.1097/aog.0000000000004057
5. MuM-PreDiCT. MuM-PreDiCT Resources: Research Documents 2021. Available: https://mumpredict.org/portfolio/shared-findings/ [Accessed 22 March 2021].
6. Knight M, Bunch K, Tuffnell D, et al. Saving Lives, Improving Mothers' Care - Lessons learned to inform maternity care from the UK and Ireland Confidential Enquiries into Maternal Deaths and Morbidity 2016-18. Oxford: National Perinatal Epidemiology Unit, University of Oxford, 2020.
7. Chen S, Zhao S, Dalman C, et al. Association of maternal diabetes with neurodevelopmental disorders: autism spectrum disorders, attention-deficit/hyperactivity disorder and intellectual disability. *International Journal of Epidemiology* 2020. doi: 10.1093/ije/dyaa212
8. Han VX, Patel S, Jones HF, et al. Maternal acute and chronic inflammation in pregnancy is associated with common neurodevelopmental disorders: a systematic review. *Translational Psychiatry* 2021;11(1):71. doi: 10.1038/s41398-021-01198-w
9. Tomson T, Battino D, Bonizzoni E, et al. Comparative risk of major congenital malformations with eight different antiepileptic drugs: a prospective cohort study of the EURAP registry. *The Lancet Neurology* 2018;17(6):530-38. doi: 10.1016/s1474-4422(18)30107-8 [published Online First: 2018/04/24]
10. Vounzoulaki E, Khunti K, Abner SC, et al. Progression to type 2 diabetes in women with a known history of gestational diabetes: systematic review and meta-analysis. *BMJ* 2020;369:m1361. doi: 10.1136/bmj.m1361
11. Beeson JG, Homer CSE, Morgan C, et al. Multiple morbidities in pregnancy: Time for research, innovation, and action. *PLOS Medicine* 2018;15(9):e1002665. doi: 10.1371/journal.pmed.1002665
12. Knight M, Tuffnell D, Kenyon S, et al. Saving Lives, Improving Mothers' Care - Surveillance of maternal deaths in the UK 2011-13 and lessons learned to inform maternity care from the UK and Ireland Confidential Enquiries into Maternal Deaths and Morbidity 2009-13. Oxford:

For peer review only - http://bmjopen.bmj.com/site/about/guidelines.xhtml

National Perinatal Epidemiology Unit, University of Oxford 2015; 2015. Available: https://www.npeu.ox.ac.uk/downloads/files/mbrrace-uk/reports/MBRRACE-UK%20Maternal%20Report%202015.pdf [Accessed 1 June 2020].
13. Williamson PR, Altman DG, Bagley H, et al. The COMET Handbook: version 1.0. *Trials* 2017;18(Suppl 3):280-80. doi: 10.1186/s13063-017-1978-4
14. CROWN. Core Outcome in Women's and Newborn Health. Available: http://www.crown-initiative.org/core-outcome-sets/ [Accessed 13 June 2020].
15. COMET initiative. Core Outcome Measures in Effectiveness Trials. Available: http://www.comet-initiative.org/ [Accessed 13 June 2020].
16. Slavin V, Creedy DK, Gamble J. Core Outcome Sets Relevant to Maternity Service Users: A Scoping Review. *J Midwifery Womens Health* 2021 doi: 10.1111/jmwh.13195 [published Online First: 2021/02/11]
17. Kirkham JJ, Davis K, Altman DG, et al. Core Outcome Set-STAndards for Development: The COS-STAD recommendations. *PLOS Medicine* 2017;14(11):e1002447. doi: 10.1371/journal.pmed.1002447
18. Kirkham JJ, Gorst S, Altman DG, et al. Core Outcome Set-STAndardised Protocol Items: the COS-STAP Statement. *Trials* 2019;20(1):116. doi: 10.1186/s13063-019-3230-x
19. Kirkham JJ, Gorst S, Altman DG, et al. Core Outcome Set–STAndards for Reporting: The COS-STAR Statement. *PLOS Medicine* 2016;13(10):e1002148. doi: 10.1371/journal.pmed.1002148
20. COMET Database. Core outcome set for studies of pregnancy affected by multimorbidity 2020. Available: https://cometinitiative.org/Studies/Details/1724 [Accessed 21 March 2021].
21. Williams K, Thomson D, Seto I, et al. Standard 6: Age Groups for Pediatric Trials. *Pediatrics* 2012;129(Supplement 3):S153-S60. doi: 10.1542/peds.2012-0055I
22. Jacob CM, Baird J, Barker M, et al. The Importance of a Life Course Approach to Health: Chronic Disease Risk from Preconception through Adolescence and Adulthood. Available: https://www.who.int/life-course/publications/life-course-approach-to-health.pdf?ua=1 [Accessed 22 April 2021].
23. Yu Y, Arah OA, Liew Z, et al. Maternal diabetes during pregnancy and early onset of cardiovascular disease in offspring: population based cohort study with 40 years of follow-up. *BMJ* 2019;367:l6398. doi: 10.1136/bmj.l6398
24. MuM-PreDiCT. Multimorbidity and Pregnancy: Determinants, Clusters, Consequences and Trajectories 2020. Available: https://mumpredict.org/ [Accessed 11 July 2020].
25. Moss N, Daru J, Lanz D, et al. Involving pregnant women, mothers and members of the public to improve the quality of women's health research. *BJOG: An International Journal of Obstetrics & Gynaecology* 2017;124(3):362-65. doi: 10.1111/1471-0528.14419
26. Nijagal MA, Wissig S, Stowell C, et al. Standardized outcome measures for pregnancy and childbirth, an ICHOM proposal. *BMC health services research* 2018;18(1):953. doi: 10.1186/s12913-018-3732-3
27. Devane D, Begley CM, Clarke M, et al. Evaluating maternity care: a core set of outcome measures. *Birth* 2007;34(2):164-72. doi: 10.1111/j.1523-536X.2006.00145.x
28. Herman D, Lor KY, Qadree A, et al. Composite adverse outcomes in obstetric studies: a systematic review. *BMC Pregnancy Childbirth* 2021;21(1):107. doi: 10.1186/s12884-021-03588-w
29. Smith SM, Wallace E, Salisbury C, et al. A Core Outcome Set for Multimorbidity Research (COSmm). *Annals of family medicine* 2018;16(2):132-38. doi: 10.1370/afm.2178
30. Ho ISS, Guthrie B, Hodgins P. A systematic review of international studies on multimorbidity measures. PROSPERO 2020 CRD42020172409 2020. Available: https://www.crd.york.ac.uk/prospero/display_record.php?RecordID=172409 [Accessed 18 March 2021].

For peer review only - http://bmjopen.bmj.com/site/about/guidelines.xhtml

31. Brouwer ME, Williams AD, van Grinsven SE, et al. Offspring outcomes after prenatal interventions for common mental disorders: a meta-analysis. *BMC Medicine* 2018;16(1):208. doi: 10.1186/s12916-018-1192-6
32. Keeley T, Williamson P, Callery P, et al. The use of qualitative methods to inform Delphi surveys in core outcome set development. *Trials* 2016;17(1):230. doi: 10.1186/s13063-016-1356-7
33. Retzer A, Sayers R, Pinfold V, et al. Development of a core outcome set for use in community-based bipolar trials-A qualitative study and modified Delphi. *PLoS One* 2020;15(10):e0240518. doi: 10.1371/journal.pone.0240518
34. Viau-Lapointe J, D'Souza R, Rose L, et al. Development of a Core Outcome Set for research on critically ill obstetric patients: A study protocol. *Obstetric medicine* 2018;11(3):132-36. doi: 10.1177/1753495x18772996
35. Braun V, Clarke V. Using thematic analysis in psychology. *Qualitative Research in Psychology* 2006;3(2):77-101. doi: 10.1191/1478088706qp063oa
36. Harman NL, Bruce IA, Callery P, et al. MOMENT--Management of Otitis Media with Effusion in Cleft Palate: protocol for a systematic review of the literature and identification of a core outcome set using a Delphi survey. *Trials* 2013;14:70. doi: 10.1186/1745-6215-14-70
37. Al Wattar BH, Tamilselvan K, Khan R, et al. Development of a core outcome set for epilepsy in pregnancy (E-CORE): a national multi-stakeholder modified Delphi consensus study. *BJOG : an international journal of obstetrics and gynaecology* 2017;124(4):661-67. doi: 10.1111/1471-0528.14430
38. Dodd S, Clarke M, Becker L, et al. A taxonomy has been developed for outcomes in medical research to help improve knowledge discovery. *Journal of clinical epidemiology* 2018;96:84-92. doi: 10.1016/j.jclinepi.2017.12.020
39. Custer R, Scarcella J, Stewart B. The Modified Delphi Technique - A Rotational Modification. *Journal of Career and Technical Education* 1999;15(2):1-10.
40. Swedish Agency for Health Technology Assessment and Assessment of Social Services. Development of a Core Outcome Set (COS) for treatment of depression during or after pregnancy (antenatal and postpartum depression). 2020. Available: https://www.sbu.se/314e [Accessed 21 March 2021].
41. Egan AM, Bogdanet D, Griffin TP, et al. A core outcome set for studies of gestational diabetes mellitus prevention and treatment. *Diabetologia* 2020;63(6):1120-27. doi: 10.1007/s00125-020-05123-6
42. Egan AM, Galjaard S, Maresh MJA, et al. A core outcome set for studies evaluating the effectiveness of prepregnancy care for women with pregestational diabetes. *Diabetologia* 2017;60(7):1190-96. doi: 10.1007/s00125-017-4277-4
43. Centre for BME Health. Toolkits. Increasing participation of Black Asian and Minority Ethnic (BAME) groups in health and social care research. Available: https://centreforbmehealth.org.uk/resources/toolkits/ [Accessed 21 April 2021].
44. National Institute for Health Research. Improving inclusion of under-served groups in clinical research: Guidance from INCLUDE project. V1.0 2020. Available: https://www.nihr.ac.uk/documents/improving-inclusion-of-under-served-groups-in-clinical-research-guidance-from-include-project/25435 [Accessed 21 April 2021].
45. Egan AM, Dunne FP, Biesty LM, et al. Gestational diabetes prevention and treatment: a protocol for developing core outcome sets. *BMJ Open* 2019;9(11):e030574-e74. doi: 10.1136/bmjopen-2019-030574
46. GRADE. GRADE Handbook: 3. Selecting and rating the importance of outcomes 2013. Available: https://gdt.gradepro.org/app/handbook/handbook.html [Accessed 14 June 2020].
47. D'Souza R, Villani L, Hall C, et al. Core outcome set for studies on pregnant women with vasa previa (COVasP): a study protocol. *BMJ Open* 2020;10(7):e034018. doi: 10.1136/bmjopen-2019-034018

For peer review only - http://bmjopen.bmj.com/site/about/guidelines.xhtml

48. Murphy MK, Black NA, Lamping DL, et al. Consensus development methods, and their use in clinical guideline development. *Health technology assessment (Winchester, England)* 1998;2(3):i-iv, 1-88.
49. Williamson PR, Altman DG, Blazeby JM, et al. Developing core outcome sets for clinical trials: issues to consider. *Trials* 2012;13(1):132. doi: 10.1186/1745-6215-13-132

For peer review only

For peer review only - http://bmjopen.bmj.com/site/about/guidelines.xhtml

Figure 1: Flowchart of COS development method

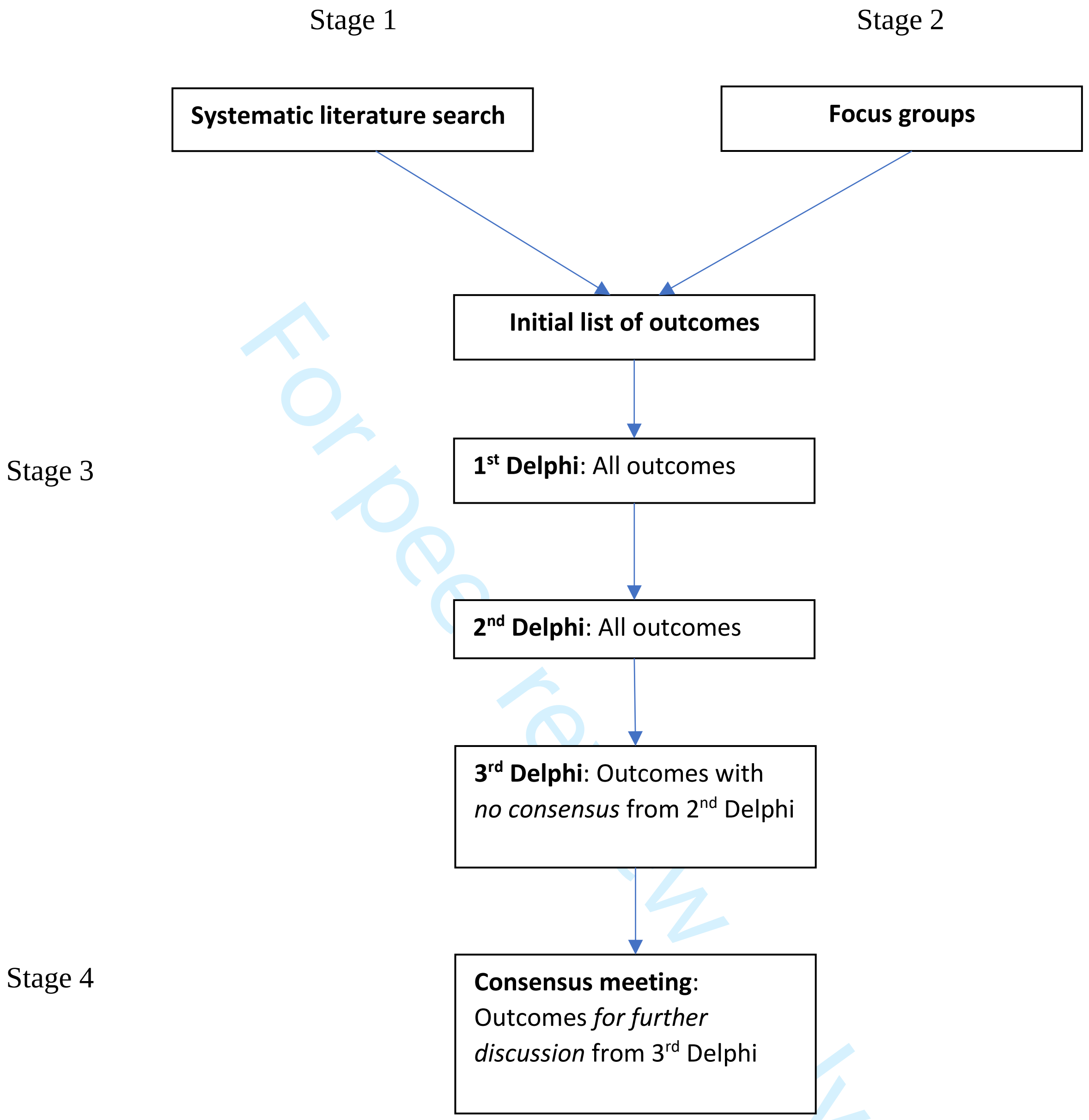

NB: For the 2nd, 3rd Delphi surveys and the consensus meeting, an aggregate score from the previous round, stratified by stakeholder groups, will be presented.

For peer review only - http://bmjopen.bmj.com/site/about/guidelines.xhtml

Figure 2: Description of patient and public involvement in the core outcome set development

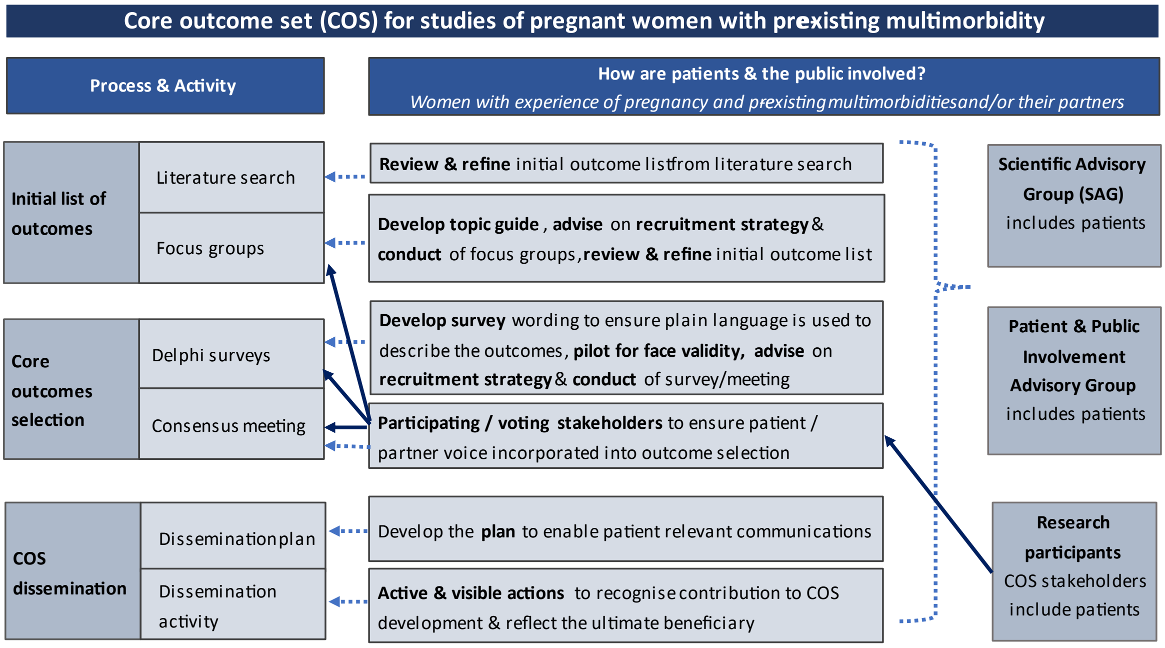

For peer review only

Appendix 1: The Core Outcome Set-STAndardised Protocol Items (COS-STAP) Statement			
**Sections**	**No**	**Items**	**Location in manuscript**
TITLE/ABSTRACT			
Title	1a	Identify in the title that the paper describes the protocol for the planned development of a COS	page 1 Title page
Abstract	1b	Provide a structured abstract	page 2 Abstract
INTRODUCTION			
Background and objectives	2a	Describe the background and explain the rationale for developing the COS, and identify the reasons why a COS is needed and the potential barriers to its implementation	page 4 Background
	2b	Describe the specific objectives with reference to developing a COS	page 4 Background
Scope	3a	Describe the health condition(s) and population(s) that will be covered by the COS	Page 5: Scope of the COS
	3b	Describe the intervention(s) that will be covered by the COS	Page 5: Scope of the COS
	3c	Describe the context of use for which the COS is to be applied	Page 5: Scope of the COS
METHODS			
Stakeholders	4	Describe the stakeholder groups to be involved in the COS development process, the nature of and rationale for their involvement and also how the	Table 1 and Table 2

		individuals will be identified; this should cover involvement both as members of the research team and as participants in the study	
Information sources	5a	Describe the information sources that will be used to identify the list of outcomes. Outline the methods or reference other protocols/papers	Page 5: Stage 1 Systematic literature search<br>Page 6: Stage 2 Focus groups
	5b	Describe how outcomes may be dropped/combined, with reasons	Page 8: Initial list of outcomes
Consensus process	6	Describe the plans for how the consensus process will be undertaken	Page 11: Consensus meeting
Consensus definition	7a	Describe the consensus definition	Page 10: Score criteria for consensus
	7b	Describe the procedure for determining how outcomes will be added/combined/dropped from consideration during the consensus process	Page 10: Score criteria for consensus
ANALYSIS			
Outcome scoring/feedback	8	Describe how outcomes will be scored and summarised, describe how participants will receive feedback during the consensus process	Page 10: 1st-3rd Delphi survey
Missing data	9	Describe how missing data will be handled during the consensus process	Page 10: 3rd Delphi survey
ETHICS and DISSEMINATION			
Ethics approval/informed consent	10	Describe any plans for obtaining research ethics committee/institutional review board approval in relation to the consensus process and describe	Page 6: Focus group<br>Page 8: 1st Delphi<br>Page 12: Ethics

		how informed consent will be obtained (if relevant)	
Dissemination	11	Describe any plans to communicate the results to study participants and COS users, inclusive of methods and timing of dissemination	Page 12: Dissemination
ADMINISTRATIVE INFORMATION			
Funders	12	Describe sources of funding, role of funders	Page 12: Funding statement
Conflicts of interest	13	Describe any potential conflicts of interest within the study team and how they will be managed	Page 12: Conflict of interest

For peer review only
